# Notes on the Use of Sulphur Preparations in Skin Diseases

**Published:** 1891-06

**Authors:** Charles Szadek

**Affiliations:** Kieff, Russia


### NOTE ON THE USE OF SULPHUR PREPARATIONS IN SKIN DISEASES.

BY CHARLES SZADEK, M. D., KIEFF, RUSSIA.

Sulphur is a remedy of great practical utility in the external and internal treatment of many skin diseases, and although it has been known by the medical profession for nearly two cenuries, it has not been very largely employed by modern physicians. My attention was attracted to this drug some time ago, as I have knowledge with paper of Dr. Unna on "Ichthyol and Resorcin in the Treatment of Skin Diseases (1886).

I have for some time employed the sulphur preparations in the form of ointment or powder in my private practice as a remedy in various skin diseases, and in many cases I have obtained very favorable and surprising results. The first case was one of long-standing rosacea in a married lady, thirty-two years old, having a family history which was negative as regarded tuberculosis, syphilis and scrofula; presented herself with a lesion on the face, which, she said, was first noticed about three years ago as small pimples near the middle of the nose. It had spread symmetrically also over the cheeks down to the mouth, passing upward to the root of the nose. The patient had been treated by some noted specialists in this section, and had not been benefited to any extent. The skin affection involved the nose and the cheeks partly ; these surfaces were all dark copper-red, with multiple nodules and pustules ; the vessels were strongly injected.

I have prescribed an ointment of precipitated sulphur, one part to eight parts of vaseline. After a fortnight's external use of the sulphur, the color of the affected skin-region became much clearer and the eruption began to show the effect of the sulpher treatment. There has not been the slightest irritation produced by the drug. In three months the disease of the skin disappeared and the patient was cured.

In the same way, and with equal success, I have treated another case of erythematous rosacea and some cases of so-called seborrhœic eczema (Unna). I have also found excellent results to follow the use of sulphur preparations in hyperidrosis, whether of the face, hands, axillæ or other regions. In cases of seborrhœa of the scalp an ointment of one part of sulphur to eight to ten parts of oil was the simplest and at the same time one of the most efficacious remedies. I have prescribed the sulphur-ointment in some cases of chronic eczema in children, and in prurigo, ichthyosis, etc., which I have been in the habit of treating by usual methods, and sometimes the results have been very gratifying and satisfactory from the use of sulphur more than from that of other remedies. In the same affections I have used the preparations of ichthyol, as a substitute for sulphur, according to the prescriptions of Unna, externally and internally, and I have also obtained very favorable results. I have not observed any injurious or disagreeable action of the remedy. On the contrary, the internal use of ichthyol, five to ten drops two or three times a day, was followed by increased appetite and powers of digestion.

I have employed the sulphur preparations as in the following prescriptions :

1. Take of

Sublimed sulphur, ½ drachm.

Salicylic acid, 8 grains.

Powdered arrowroot, ½ ounce.

Mix.

2. Take of

Sublimed sulphur, ½ drachm.

Almond-oil,

Glycerine, aa 3 ounces.

Mix.

3. Take of
Sublimed sulphur, 2 drachms.
Ætheris sulphuris,
Spirits vini rectifi, aa 2 ounces.
Mix. Sig. Shake well and mop over the surface.

4. Take of
Sublimed sulphur, 2 scruples.
Vaseline or ointment of benzoated oxide of zinc, 1 ounce.

5. Take of

Ichthyol (sulpho-ichthyolate of sodium or ammonium),

Spirits vini rectifi, aa 1 ounce.

M. D. S. Five to ten drops two or three times a day.

6. Take of

Ichthyol, 2 scruples.

Powdered oxide of zinc,

Powdered arrowroot, aa 2 ounces.

Vaseline, ½ ounce.

Mix. f. pasta.

7. Take of

Ichthyol, ½ to 1 drachm.

Vaseline, or ointment of benzoated oxide of zinc, ½ ounce.

CHANCRES OF THE FINGERS.

An excellent clinical monograph on the subject of chancres of the fingers has been published in *N. Y. Medical Record*, January 17, 1891, by R. W. Taylor, accompanied by a chromo-lithograph. He says that chancres of the fingers contracted in operations, examinations and manipulations are not uncommon among obstetricians, surgeons and midwives, and they are occasionally seen upon the hands of some luckless laymen as the result of their handling or lasciviously fondling the infected genitals of some female. They are also observed in dentists, contracted in operations on the mouths of syphilitic patients. They may be seated upon the finger pulp or around the nail. Syphilitic finger infection has been seen in children and women as a result of picking or dressing the hard chancre of a second party. Finger chancres as a result of a bite by a syphilitic person are also occasionally seen. Chancres of the knuckles and fingers have been known to follow a blow delivered upon the mouth of a person suffering from specific lesions. These chancres are prone to form at some part of the nail margin, also on the sides and on the pulp of the finger and along its continuity. In the greater number of cases they are due to the infection of a small fissure, a chap, an excoriation or a cut, or to the lodgement of the virus between the nail and its tegumentary fold. Any skin disease of an inflammatory or hyperplastic nature situated upon the fingers or hands may afford a port of entry for syphilitic infection. For clinical purposes we may divide chancres of the fingers into four forms, as follows: (1) The scaling papule or tubercle; (2) the excoriated or exulcerated nodule or mass; (3) the fungating chancre; and (4) the panaritium form chancre. First form of digital chancre (papule or tubercle form) is the newest and simplest in character, and is the rarest of all forms observed on these members. It is usually found upon the dorsal surface of a phalanx, and occasionally it is seated upon the palmar surface or the sides of the fingers. It usually begins on the site of a slight cut, an excoriation or a fissure. The traumatic lesion may or may not heal and disappear. The chancre begins as a small red spot, which may or may not be scaly. This spot usually grows quite promptly, and in about a fortnight a round or oval papule or papulo-tubercle is formed. Thus it may remain in an indolent, slightly scaly state or it may extend. The scaling papule may exist for weeks, or two or three months, and then it sinks down and disappears, leaving a thickened, more or less purplish or brownish red pigmented site, which may or may not be the seat of induration or of more or less atrophy. The excoriated or exulcerated form of digital chancre is usually found toward the end of the finger, and to have its origin in some lesion of the nail margin, which may be an excoriation or fissure, a hangnail or even some slight separation between the skin and the nail inappreciable to the naked eye. The exulcerated nodule usually begins as a small pustule or as a minute, red excoriation of varying shape. This latter lesion increases quite rapidly in area and in height, and is, in its early days, indolent and painless. It may reach only to small size, but usually the hyperplasia is luxuriant. Full development may occupy a period of two to four weeks. The shape of the lumpy mass depends on its situation. If seated on one side of the finger it may jut out from one-half to one inch. If both sides of the finger are involved, the mass may become as large as a horse chestnut. The course of this chancre depends entirely upon the manner in which it is treated. If kept free from irritation and at rest, it will slowly subside, but if irritated its duration is indefinite. The author has seen one case which has existed for nearly a year. Their chronicity is an important matter, for it carries with it danger of infection to others. Third form of digital chancre, the fungating ulceration, is a not uncommon one, and begins in a moist or dry red spot, or a pustule upon the seat of some traumatism; then, generally, granulations begin to spring up and the surface of the chancre becomes fungating. They are usually of a dull bluish or purplish red color, and may also resemble in some cases condylomata lata, which have become vegetating. These fungating chancres sometimes run an indolent course, showing little, it any, tendency to healthy action. Under proper treatment the granulations give place to a healthy healing surface. There is commonly more or less deformity left by these chancres. Either the nail is more or less permanently destroyed or a retractile and perhaps fibered red or purplish red scar is left which constitutes a a permanent deformity. Phlebitis and lymphangitis of a new, even decided type, may be an accompaniment of this chancre at some period of its existence.

The panaritium-like chancre of the finger is not very common, and is seen with rather more frequency than the scaling chancre of the finger, and less frequently than the ulcerated nodule or the fungating mass.

This form of chancre of the fingers usually begins on the integument of the nail margin or in a cut, a fissure, a hangnail, or in some simple inflammatory lesion, which confines itself pretty closely to the nail sulcus, along which it creeps and develops. With the increase of the specific perionychia the distal phalanx begins to swell, and the finger becomes distorted. As the hypertrophic process increases ulceration is usually seen around the nail, to which it may be limited in a crescent-like form. This ulceration usually has an unhealthy, foul surface, secretes a copious, fœtid pus, and may be covered in whole or in part with a brownish black or green necrotic film or membrane. The nail rapidly becomes darker, until it is detached as a black looking, foul mass, under which the ulceration has extended. In this state of full development this panaris-like chancre may remain for a long period without any tendency to heal, and sometimes without any evidence of exacerbation. Under proper treatment, however, it will slowly subside, and in the course of several months the end of the finger may be nearly normal in size. Sometimes it becomes much atrophied and pointed, and the seat of an irregular cicatrix. In most cases the nail is impaired, sometimes wholly destroyed, and sometimes only partially so. This form of finger chancre may be mistaken for non-specific panaritium, and for the early and later perionychiæ of syphilis. Accompanying the panaritium-like chancre a mild or severe phlebitis or lymphangitis may occur. The author reports three cases of digital chancre, in which pyemic and septic infection occurred. In two cases, it is probable that the agent of infection was the streptococcus pyogenes of Rosenbach. The history of the third case is very interesting and peculiar. In this case there was no pre-existent chancre, but syphilitic and septic infection took place simultaneously through the entry of the secretion of a phagedenic chancre into a minute healthy wound on the woman's finger.

The fact of the not uncommon occurrence of chancres upon the fingers of surgeons, physicians, and midwives suggests to us the danger of syphilitic infection to their patients. Cases of syphilis transmitted by finger infection in medical and obstetrical practice are sufficiently common and well attested to warrant us in a consideration of the question of prophylaxis. It is evident that surgeons afflicted with syphilis are much less liable to infect their patients than obstetricians, particularly in these days of thorough asepsis and antisepsis. The period of greatest danger from finger infection by medical men is in the early days of the chancre, when it is small and well, and its nature is not suspected. In this period it is very little, if at all painful; it has not an angry or an inflamed look, but, on the contrary, present a benign, even insignificant, appearance.

(*Medical Record*, New York, 1891, I, 3, pages 69–73).

