# Erratum

**Published:** 1891-06

**Authors:** 


### CONTENTS.

ORIGINAL COMMUNICATIONS—

Observations on Koch's Lymph. By Joseph Jones, M. D., New Orleans........

Suggestions about Abdominal and Pelvic Surgery. By Wm. H. Wathen, M. D., Louisville, Ky........ 199

When Should We Interfere in Threatened Puerperal Convulsions? By Edward H. Richardson, M. D., Atlanta........ 207

Perityphlitis. By Joseph McEvoy, M. D., Atlanta........ 214

A Plea for Conservative Minor Gynecology. By R. R. Kime, M. D., Atlanta........ 218

An Interesting Operation for Ovarian Cyst. By Jno. R. Bradfield, M. D., and Fred'k M. Reeves, M. D., Alvord, Texas........ 223

*Notes on the Use of Sulphur Preparations in Skin Diseases. By Charles Szadek, M. D., Kieff, Russia........ 225

SOCIETY REPORTS—

Gynecological and Obstetrical Society of Baltimore........ 231

Tennessee State Medical Society........ 241

CORRESPONDENCE—

Our New York Letter........ 244

EDITORIAL—

Meeting of The American Medical Association........ 251

Items........ 253, 254, 255, 256

